# Seed germination response to high temperature and water stress in three invasive Asteraceae weeds from Xishuangbanna, SW China

**DOI:** 10.1371/journal.pone.0191710

**Published:** 2018-01-24

**Authors:** Xia Yuan, Bin Wen

**Affiliations:** 1 Center for Integrative Conservation, Xishuangbanna Tropical Botanical Garden, the Chinese Academy of Sciences, Mengla, Yunnan, China; 2 University of the Chinese Academy of Sciences, Beijing, China; Brigham Young University, UNITED STATES

## Abstract

*Crassocephalum crepidioides*, *Conyza canadensis*, and *Ageratum conyzoides* are alien annuals naturalized in China, which produce a large number of viable seeds every year. They widely grow in Xishuangbanna, becoming troublesome weeds that compete with crops for water and nutrients. As seed germination is among the most important life-stages which contribute to plant distribution and invasiveness, its adaptation to temperature and water stress were investigated in these three species. Results showed that: (1) These three species have wide temperature ranges to allow seed germination, i.e., high germination and seedling percentages were achieved between 15°C and 30°C, but germination was seriously inhibited at 35°C; only *A*. *conyzoides* demonstrated relative preference for warmer temperatures with approximately 25% germination and seedling percentage at 35°C; (2) light was a vital germination prerequisite for *C*. *crepidioides* and *A*. *conyzoides*, whereas most *C*. *canadensis* seeds germinated in full darkness; (3) Although all three species have good adaptation to bare ground habitat characterized by high temperatures and water stress, including their tolerance to soil surface temperatures of 70°C in air-dried seeds, *A*. *conyzoides* seeds exhibited higher tolerance to both continuous and daily periodic high-temperature treatment at 40°C, and to water restriction (e.g., ca. 65% seeds germinated to -0.8 MPa created by NaCl), which is consistent with their field behavior in Xishuangbanna. This study suggests that seed high-temperature tolerance contributes to the weed attributes of these three species, and that adaptation to local micro-habitats is a critical determinant for invasiveness of an alien plant.

## Introduction

Biological invasion caused tremendous environmental and agriculture damage worldwide, which has been listed as one of the most serious problems faced by human beings [1,2]. Asteraceae is one of the angiosperm families producing many of the most troublesome invasive weeds, partly because they are prolific seed producers with the capability to produce small but numerous viable seeds every year, in addition to wind-assisted seed dispersal, rapid growth, a short juvenile period, and high reproductive efforts, such as *Crassocephalum crepidioides* (commonly known as hawksbeard, native to tropical Africa), *Conyza canadensis*, and *Ageratum conyzoides* (both native to North America, and commonly known as horseweed and goatweed, respectively, [3–5]). These three species were found to have appeared in China during the 19th and 20th Century [6]. They have now naturalized as annual weeds, and are aggressive, noxious and abundant throughout tropical and subtropical China [6].

In a review, Donohue et al. concluded that seed germination was an important developmental phase change in the plant life cycle, which plays critical roles in seedlings establishment and environmental adaptation [7]. Previous studies have frequently correlated seed germination traits to plant invasiveness, e.g., alien invasive species was found to germinate more rapid, higher, and more successful across more varied environmental conditions, and in response to increased resource availability than congeneric native/noninvasive [8,9]. These three invasive Asteraceae weeds are popular on bare ground and along roadsides in Xishuangbanna, SW China. Wen [10] proposed that plant species growing in such habitats should have strategies to adapt to extreme fluctuations in temperature and moisture conditions there. This study aimed to firstly test this hypothesis. From a literature review, we found that the distribution range of *Ageratum conyzoides* is more southern than the other two species, although they all have a very wide distribution in China, and greatly overlap one another [11]. We assumed that this trait may be reflected in their seed germination.

Knowledge of the influence of climatic and edaphic factors on the germination and emergence behavior of seeds or other propagules is important for understanding the distribution and biology of a species, as well as determining its management practices [12,13]. Due to their wide distribution and marked harmfulness as weeds, these three invasive species have been investigated in detail [14–24], but very little is known about their adaptation to invaded habitats. To increase our knowledge on their advantages and limitations as invasive species, the present study investigated the responses of seed germination in *C*. *crepidioides*, *C*. *canadensis*, and *A*. *conyzoides* to temperature and water stress.

## Materials and methods

### Plants

In Xishuangbanna, *C*. *crepidioides*, *C*. *canadensis*, and *A*. *conyzoides* have a long flowering period, with mature achenes (hereafter called seeds, although technically they are dry fruits) available from March to November, with most seeds maturing from August to December, which are then blown off by wind.

### Seed materials

Seeds used in this study were collected from Menglun, where Xishuangbanna Tropical Botanical Garden, the Chinese Academy of Sciences (21°55ʹ N, 101°15ʹE) is located, in August 2016. They were harvested from a number of plants and pooled. After collection, seeds were air-dried in ambient conditions for a few days, then debris removed, seed weight and initial moisture content determined, and initial viability assessed using methods described below. The remaining seeds were sealed in polyethylene bags and kept at 15°C for approximately 1–5 months before the following experiments were performed from October 2016 to April 2017.

### Experimental arrangements

As *C*. *crepidioides*, *C*. *canadensis*, and *A*. *conyzoides* are weeds growing only on open fields, i.e., habitats which have characteristics of high light intensity and enhanced fluctuations in temperature and water availability, we were wondering whether they have specific adaptations to these stresses. According to our previous study [25,26], the following series of experimental designs were carried out in this study to investigate the effects of high temperature and water stress on seed germination and seedling in these three invasive species.

#### Effects of incubation temperature and light on seed germination

The objective of this study was to determine the required temperature and light regime for seed germination of the three species. Germination was performed by sowing seeds on 1% water agar in Petri dishes, and incubating them at constant temperatures from 10°C to 40°C, and also at an alternating temperature of 18/28°C, with a 12 h photoperiod of 25 μmol m^-2^ s^-1^ irradiance provided by white fluorescent lamps. For the darkness treatment, dishes with seeds were wrapped in double layer of aluminum foil to prevent any light penetration. After a 40 d incubation, seeds that failed to germinate in the dark were moved to 25°C in light for another 40 d incubation to test seed viability.

#### Effects of high-temperature shock on quiescent seeds

In order to investigate seed tolerance to extreme high temperature, a water bath was used to make the temperature regime required in this experiment [10]. Two triangular flasks containing approximately 350 seeds each were applied at each testing temperature, ranging from 30°C to 95°C, for each species. The seeds in one flask were kept air-dried during heating, whereas a few drops of deionized water were added to the other flask to moisten the seeds half an hour before heat treatment. After heating for half an hour at the indicated experimental temperature, the seeds were immediately sown on 1% water agar for viability assessment.

#### Effects of continuous heat treatment on seed viability

Referring to the previous study [10], 40°C was used to determine the response of investigated seeds to continuous and periodic heat stress treatment in the present study. Seeds were sown on water agar as described above, and placed in an incubator at 40°C firstly. After heat treatment for a given period, up to 216 h, Petri dishes containing seeds were sampled, retraced from heat stress, and placed in an incubator at 25°C to check seed viability.

#### Effects of periodic high temperature on seed germination

As our previous description [10], seeds sown on 1% agar were alternatively incubated at 40°C and 25°C with varying warm periods to investigate the effects of increased daily heat duration on germination in this experiment.

#### Effects of water restriction on seed germination

NaCl and polyethylene glycol (PEG) 8000 were used to create equal water potential gradients between -0.05 and -1.0 MPa, according to Lang [27] and Michel [28], respectively, with an addition of deionized water as the control (0 MPa). Seeds sown on filter paper discs moistened with testing solutions in Petri dishes were incubated at 25°C in light for germination. In order to reduce moisture loss during the experimental period, Petri dishes for the same treatment were sealed in a resealable double-clear plastic bag in this experiment. Twice a week, seed germination was scored, with simultaneous replacement of filter papers and testing solutions. Six weeks later, the non-germinated seeds were removed to filter papers moistened with deionized water to check if the seeds were still viable.

#### Effects of dehydration treatment on seed viability during germination

An imbibition-dehydration treatment was designed to mimic the effects on seed viability of huge changes in soil water content usually happened on sunny days after a rainstorm. For this purpose, the seeds were firstly sown on filter paper discs moistened with deionized water in Petri dishes, and incubated at 25°C for different durations, from 3 h to 48 h, and then air-dried in a dry room (set at 50% RH and 15°C) for 72 h, with an additional germination scoring just before drying. After this dehydration interruption, they were watered again and returned to 25°C for viability check. Seed germination was checked twice a week, with deionized water replenished simultaneously when necessary.

### Seed viability and germination assessment

Experiments of 50 seeds × 6 replicates per species were designed for each treatment in the whole study. Seed germination was scored once a week for at least 6 weeks, unless stated otherwise. Those protruded visible radicles were considered to have germinated, or survived the stress experiments; and those had a cotyledon grown were removed (seedling). A simple pressure test was applied to assess viability of the non-germinated seeds: white, firm embryos were considered as viable, and brown, soft embryos were nonviable [29].

### Data analysis

Because germination data expressed as percentages lacked normal distribution, a generalized linear model (glm) was used to analyze seedling and germination or survival of the seeds with a binomial error distribution and logit-link function. Seedling and germination or survival percentages were separately taken as dependent variables for data analysis. Treating species and all applied environmental factors as fixed effects, interactions between factors were also evaluated. Germination data at a constant temperature of 25°C was included as a control in analysis of the effect of continuous and periodical heating treatment. All analyses were done with the statistical programming language R (v.3.3.3) [30].

## Results

### Effects of incubation temperature and light on seed germination

**Fig 1:**
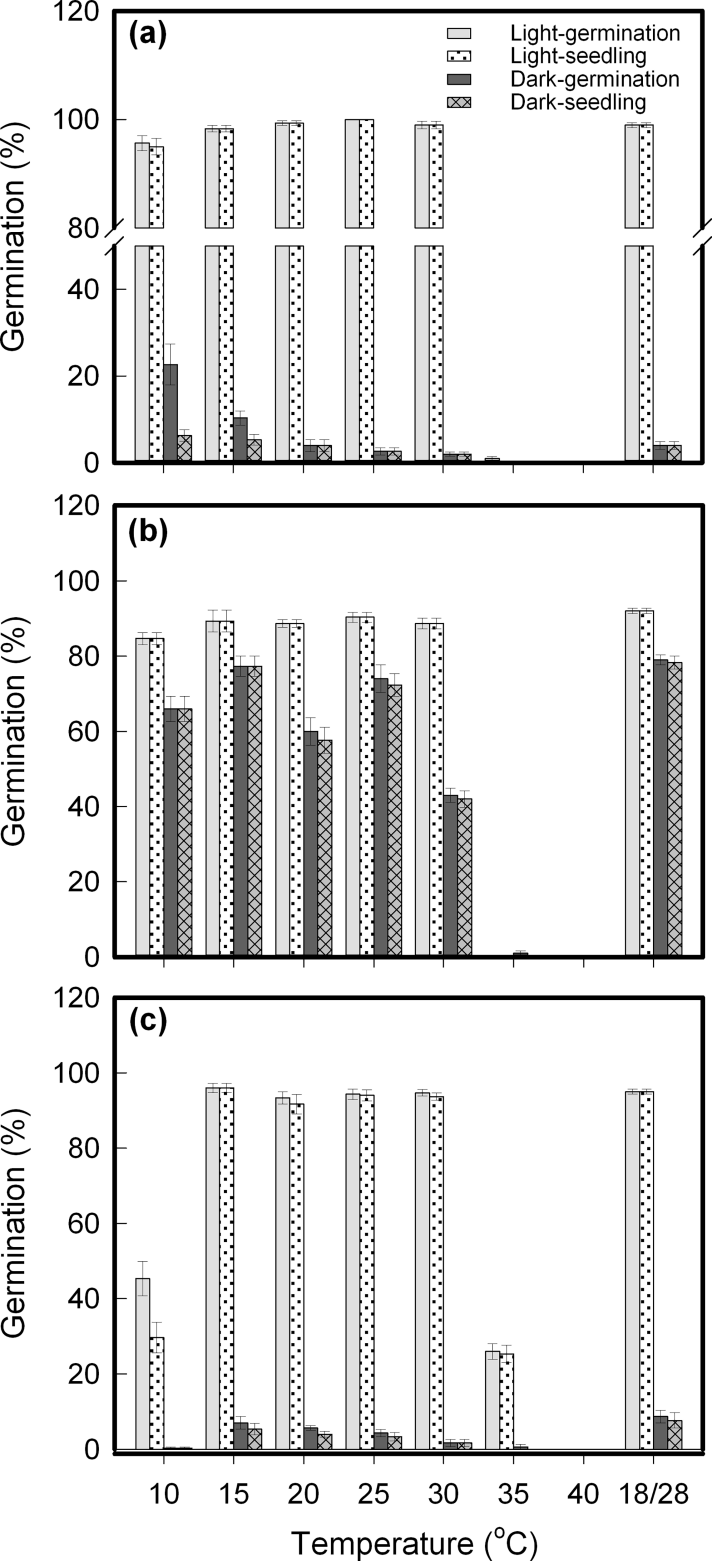
Changes in seed germination as affected by incubation temperatures and light. **a.** *Crassocephalum crepidioides*; **b.** *Conyza candensis*; **c.** *Ageratum conyzoides*. Seeds sown on 1% agar were incubated at constant temperature from 10°C to 40°C, and at 18/28°C, with periodic illumination **(light)** or under full dark **(dark)**. Germination and seedling percentages are expressed as means±SE of six replicates of 50 seeds.

It was found that germination occurred in these three species over a wide temperature range, with species, temperature, light, and their interactions all showing a significant interactive effect (Table 1). Under daily periodical light, most seeds (≥ 85%) germinated at 15°C to 30°C—even at 10°C, but only for *C*. *crepidioides* and *C*. *canadensis—*whereas germination was seriously inhibited at 35°C and above (Fig 1). *A*. *conyzoides* demonstrated some preference for high temperatures compared to *C*. *crepidioides* and *C*. *Canadensis*, which had lower germination at 10°C but approximately 25% seeds emerged at 35°C (Fig 1C), whereas germination hardly occurred in the other two species at this high temperature (Fig 1A and 1B).

**Table 1:** Summary of the generalized linear models to test the variables affecting seed seedling and germination or survival of the seeds.

Experiment	Effect	Seedling				Germination/Survival		
		Estimate±SE		*Z*-value	*P*-value	Estimate±SE	*Z*-value	*P*-value
^1^**Incubation temperature and Illumination**	Species	-0.643±0.178		-7.211	<0.001	-1.011±0.180	-5.615	<0.001
	Illumination	1.245±0.239		5.198	<0.001	1.416±0.243	5.826	<0.001
	Temperature	-0.157±0.078		5.198	<0.05	-0.187±0.078	-2.398	<0.05
	S×I	1.036±0.120		-1.998	<0.001	1.102±0.122	9.021	<0.001
	S×T	0.083±0.037		8.581	<0.05	0.143±0.037	3.872	<0.001
	I×T	0.128±0.047		2.243	<0.01	0.094±0.048	1.957	0.0503
	S×I×T	-0.158±0.023		2.673	<0.001	-0.170±0.023	-7.239	<0.001
**High-temperature tolerance**	Species	-1.021±0.149		-6.853	<0.001	-0.976±0.150	-6.472	<0.01
	Hydration status	-1.657±0.206		-8.008	<0.001	-1.495±0.210	-7.119	<0.001
	Temperature	-0.221±0.038		-5.713	<0.001	-0.221±0.038	-5.698	<0.001
	S×H	0.338±0.090		3.722	<0.001	0.276±0.091	3.008	<0.01
	S×T	0.093±0.017		5.409	<0.001	0.097±0.017	5.625	<0.001
	H×T	-0.106±0.026		-4.016	<0.001	-0.112±0.026	-4.252	<0.001
	S×H×T	-0.043±0.012		-3.560	<0.001	-0.042±0.012	-3.503	<0.001
^2^**Continuous heat treatment**	Species	-0.190±0.066		-2.856	P<0.01	-0.348±0.069	-5.024	P<0.001
	Heating duration	-0.197±0.016		-12.268	P<0.001	-0.234±0.016	-14.013	P<0.001
	S×H	-0.062±0.007		-8.127	P<0.001	-0.049±0.007	-6.330	P<0.001
^2^**Periodic heat treatment**	Species	-0.829±0.070		-11.791	P<0.01	-0.724±0.071	-10.089	P<0.001
	Heating duration	-0.219±0.024		-8.999	P<0.001	-0.124±0.025	-4.908	P<0.001
	S×H	0.023±0.010		2.171	P<0.05	-0.004±0.010	-0.411	0.681
**Water availability**	Species	0.361±0.160		2.260	<0.05	0.952±0.156	6.073	<0.001
	Reagent	0.630±0.222		2.830	<0.01	1.550±0.225	6.875	<0.001
	Water potential	-0.115±0.039		-2.913	<0.01	0.151±0.039	3.874	<0.001
	S×R	-0.345±0.100		-3.435	<0.001	-0.706±0.100	-7.059	<0.001
	S×W	-0.067±0.018		-3.644	<0.001	-0.174±0.018	-9.641	<0.001
	R×W	-0.092±0.025		-3.647	<0.001	-0.178±0.025	-7.088	<0.001
	S×R×W	0.022±0.011		1.931	0.053	0.075±0.011	6.533	<0.001
**Desiccation interruption**	Species	0.037±0.056		0.672	0.5019	0.075±0.057	1.310	0.190
	Imbibition time	-0.144±0.014		-10.015	P<0.001	-0.142±0.014	-9.814	P<0.001
	S×I	0.017±0.006		2.531	P<0.05	0.015±0.006	2.292	P<0.05

^1^The alternative temperature of 18/28°C was not included in this analysis

^2^Germination data at constant temperature of 25°C was included in these analyses as control (0 h at 40°C).

There were obvious differences in light requirement for germination among these species. Germination in the dark was rare for *C*. *crepidioides* and *A*. *conyzoides* (Fig 1A and 1C), but 90% of non-germinated seeds germinated after being transferred to light conditions at 25°C during further incubation (data not shown), indicating that they have photoblastic seeds, i.e., seeds showing phytochrome-mediated germination. In contrast, *C*. *canadensis* showed a different behavior, a relative germination percentage of 50–85% was achieved in the dark compared to light germination, depending on incubation temperatures (Fig 1B).

### High-temperature tolerance of quiescent seeds

**Fig 2:**
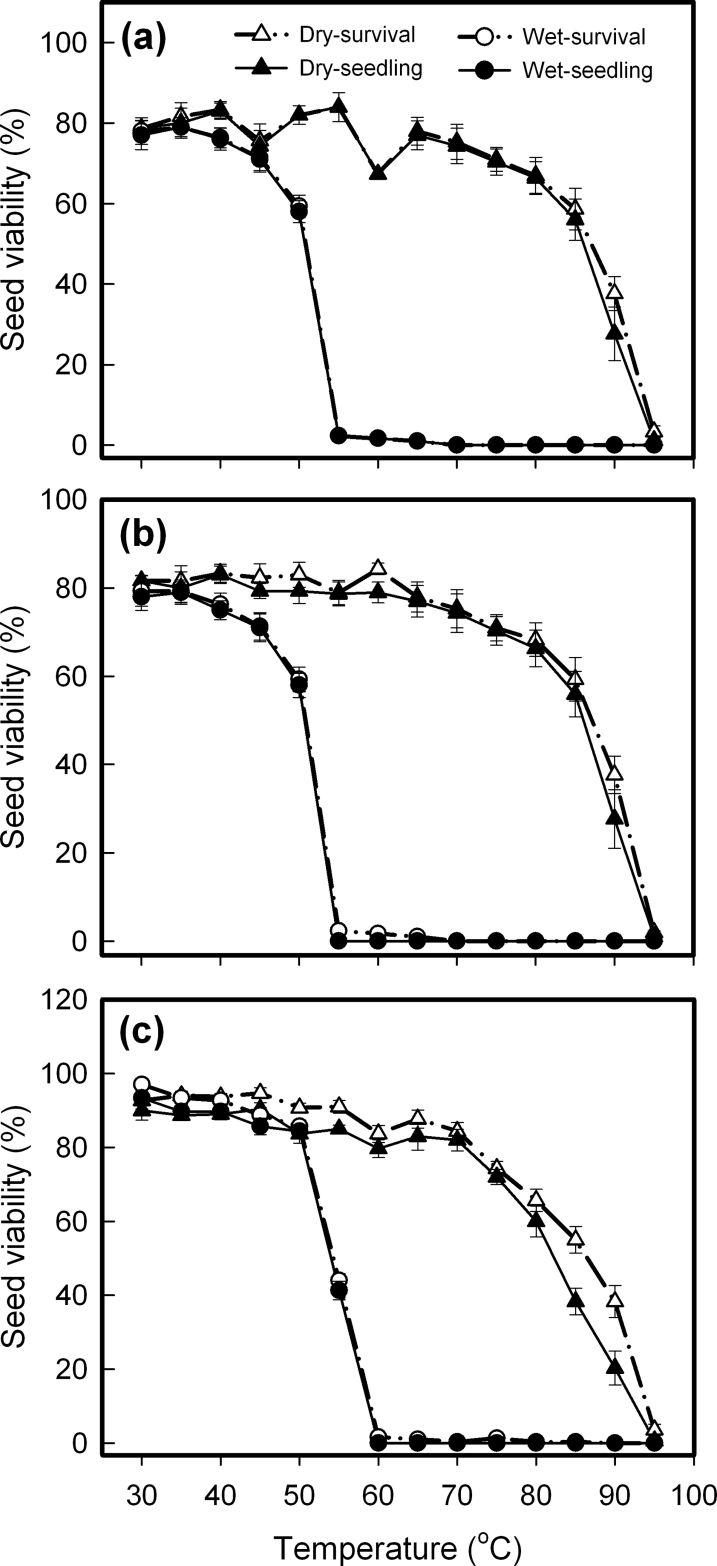
Changes in viability of air-dried (dry) and imbibed (wet) seeds as affected by 30-min heat shocks at temperatures from 30°C to 95°C. **a.** *Crassocephalum crepidioides*: **b.** *Conyza candensis*; **c.** *Ageratum conyzoides*. Survival and seedling percentages are expressed as means±SE of six replicates of 50 seeds.

Species, seed hydration, testing temperature, and their interaction all significantly affected seed viability, assessed by both survival and seedling percentage (Table 1). These three species all had in common a wide discrepancy between their viability curves for air-dried and imbibed seeds. All imbibed seeds were killed after being heated for 30 min at 55°C (for *C*. *crepidioides* and *C*. *canadensis*) or 60°C (for *A*. *conyzoides*) and above, whereas heat treatment at 70°C hardly damaged air-dried seeds, with approximately 30% surviving even up to 90°C (Fig 2).

### Effects of continuous heat treatment on seed germination

**Fig 3:**
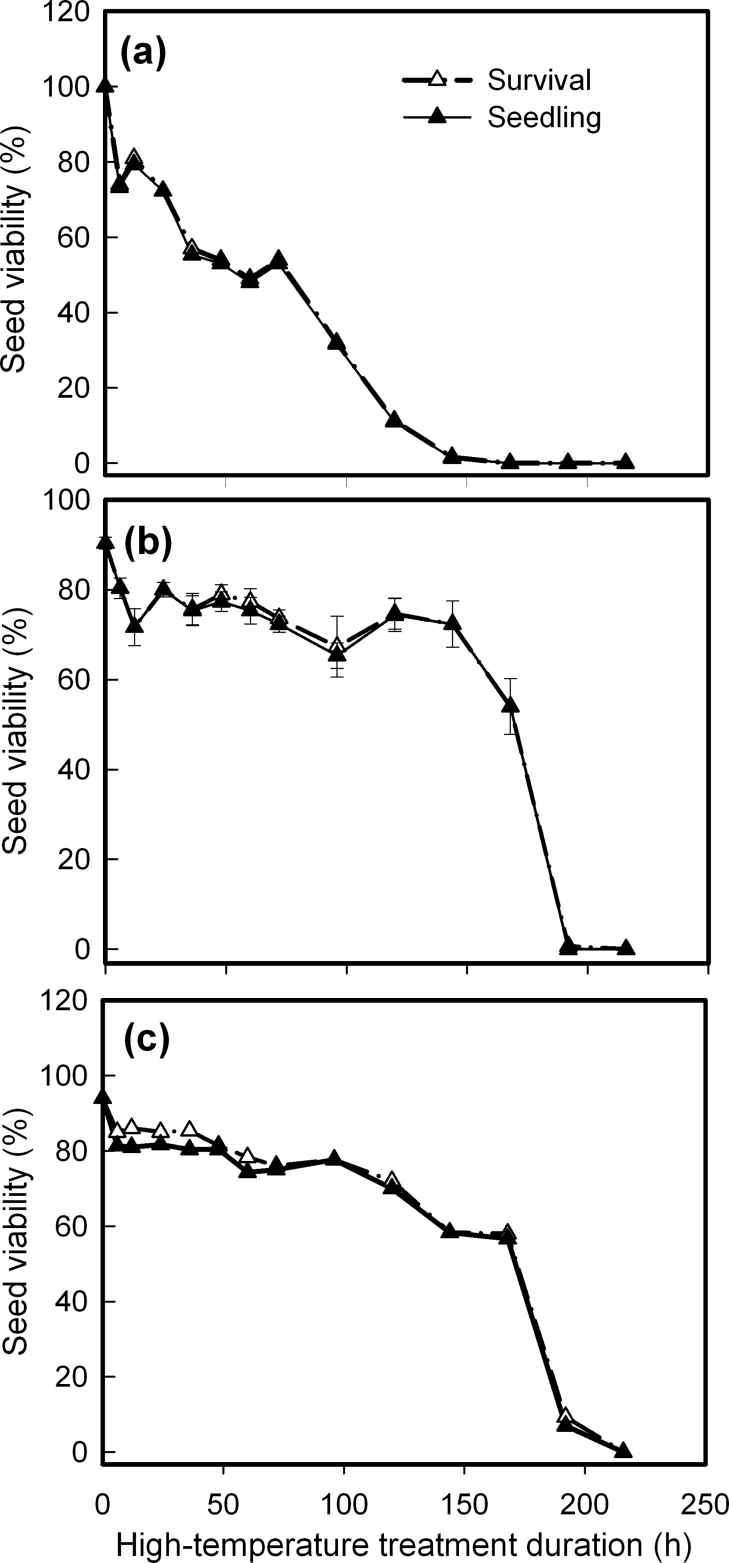
Changes in seed viability as affected by continuous high-temperature stress. **a.** *Crassocephalum crepidioides*; **b.** *Conyza candensis*; **c**. *Ageratum conyzoides*. Seeds sown on 1% agar were subjected to heat shock at 40°C for indicated durations, and incubated at 25°C after they were released from stress. Survival and seedling percentages are expressed as means±SE of six replicates of 50 seeds.

*C*. *crepidioides* demonstrated a different response model to continuous heat treatment than the other species, whose seedling and survival percentage decreased linearly as heat treatment duration increased and nearly all seeds were killed after 6-days’ heating at 40°C, whereas 6-days’ treatment caused no or only a small reduction to *C*. *canadensis* and *A*. *conyzoides* seed viability; however, after that, they lost viability abruptly (Fig 3). Species, heating duration and their interaction all had significant effects on results (Table 1). Obviously, *C*. *crepidioides* seeds had higher sensitivity to continuous high temperature than *C*. *canadensis* and *A*. *conyzoides* seeds. Only a small portion of *A*. *conyzoides* seeds survived after 8-days’ treatment (Fig 3).

### Effects of periodic high temperature on seed germination

**Fig 4:**
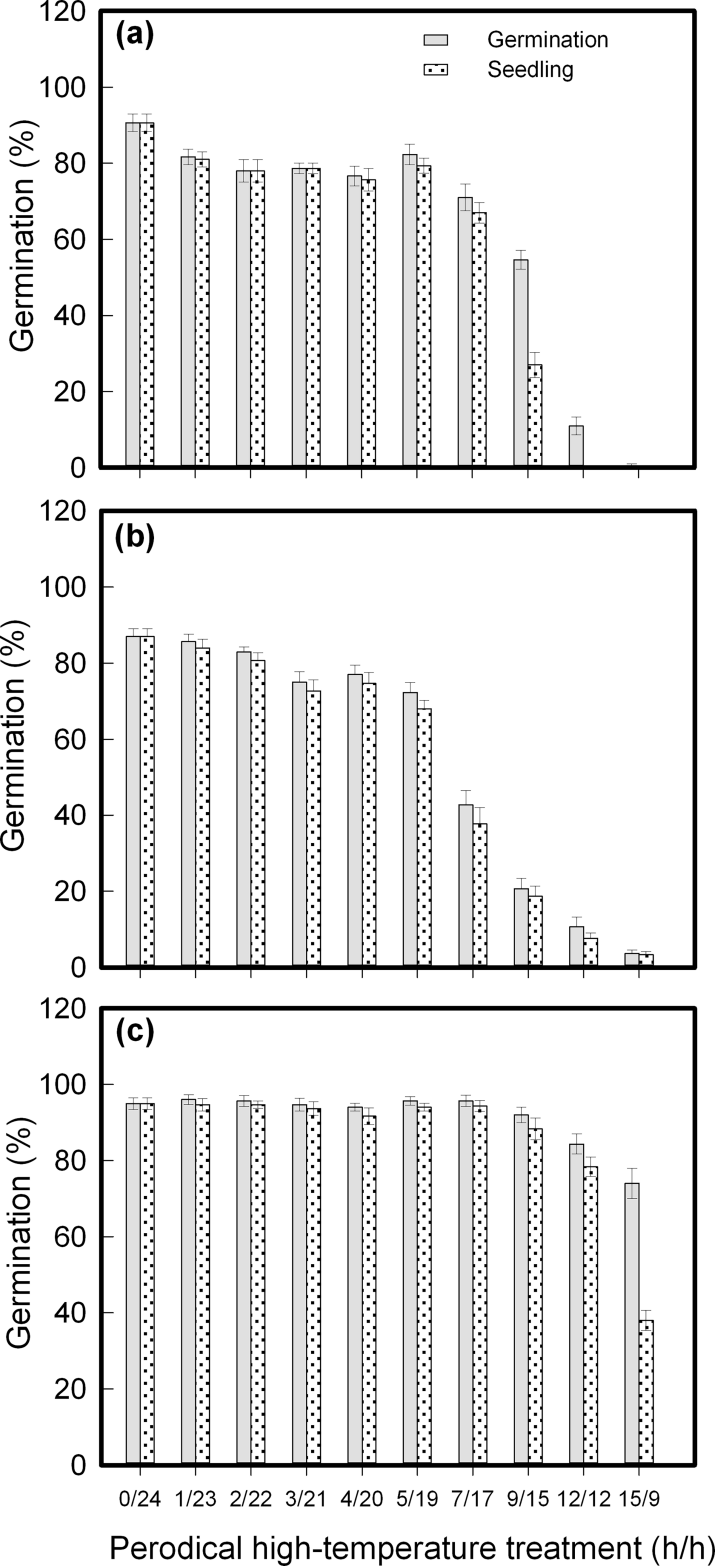
Changes in seed germination as affected by daily periodic high-temperature stress. **a.** *Crassocephalum crepidioides*; **b.** *Conyza candensis*; **c.** *Ageratum conyzoides*. Seeds sown on 1% agar were subjected to 40°C and 25°C (h/h) alternately. Germination and seedling percentages are expressed as means±SE of six replicates of 50 seeds.

These three species differ from one another in their response to daily periodic high-temperature treatment. Among them, *C*. *canadensis* is the most sensitive; *A*. *conyzoides*, the most tolerant; and *C*. *crepidioides*, intermediate. Daily heat treatments for up to 5 h at 40°C affected neither germination nor seedling; *C*. *canadensis* had a threshold at 7 h, which halved its germination and seedling percentage. *C*. *crepidioides* and *A*. *conyzoides* also had this threshold, but at 9 and 15 h, respectively (Fig 4). At their threshold points, seedling of *C*. *crepidioides* and *A*. *conyzoides* was much lower than their germination percentage, suggesting that many seeds failed to form seedlings though germinated (Fig 4).

### Effects of water availability on seed germination

**Fig 5:**
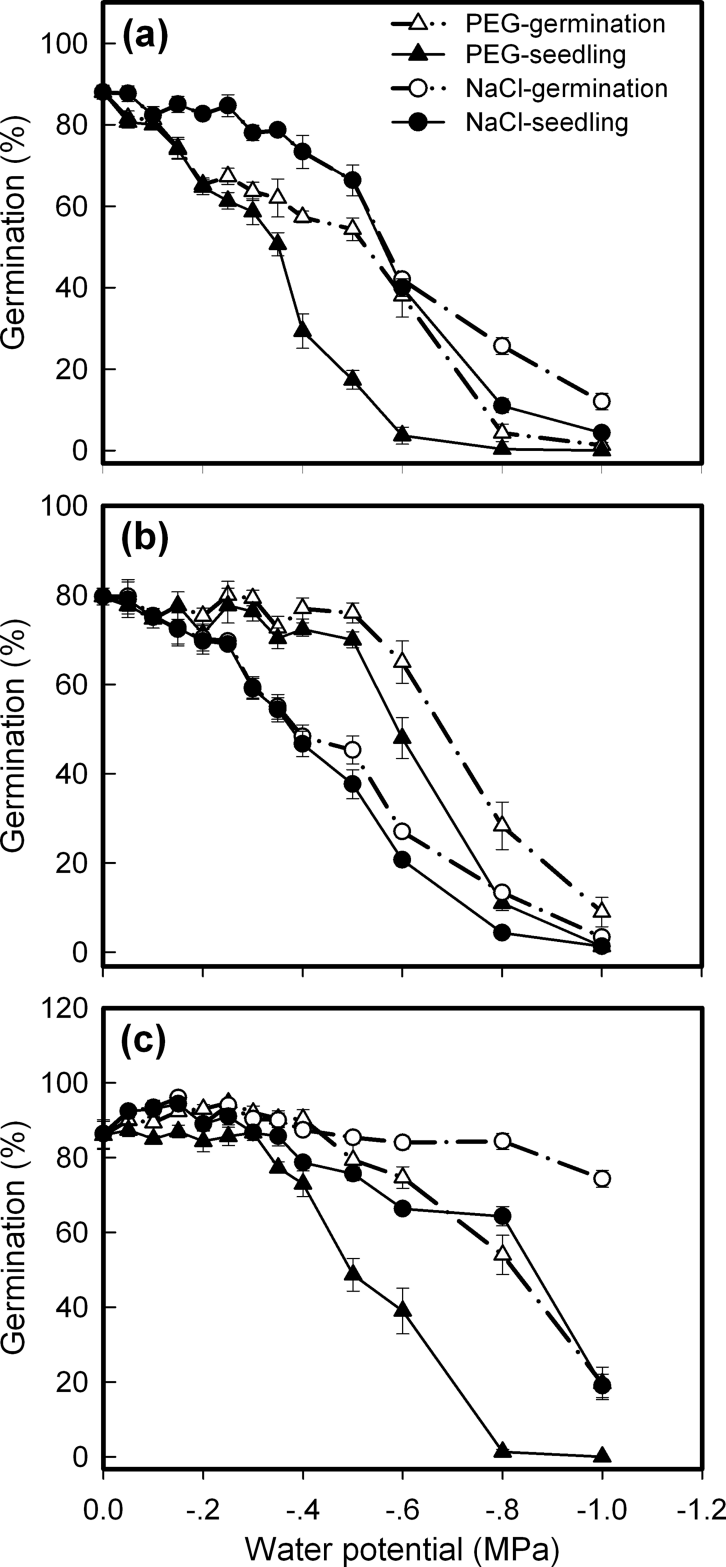
Changes in seed germination as affected by water potentials. **a.** *Crassocephalum crepidioides*; **b.** *Conyza candensis*; **c.** *Ageratum conyzoides*. Seeds were incubated at 25°C on filter papers moistened with osmotic solutions. Water potentials were created by **PEG** 8000 and **NaCl**. Germination and seedling percentages are expressed as means±SE of six replicates of 50 seeds.

This study found marked differences in seed germination under water stress (significant factors were: species, water potential, reagent, and their interactions between factors, except for S × R × W for seedling, Table 1). All three species responded to water stress, with obvious germination inhibit under water potentials lower than -0.5 MPa, but they differed in their sensitivity. Comparatively, *C*. *crepidioides* is the most sensitive and *A*. *conyzoides*, the most tolerant. For example, the highest seedling percentage achieved at water potential of -0.6 MPa was ca. 40%, 50% and 65% for *C*. *crepidioides*, *C*. *canadensis* and *A*. *conyzoides*, respectively. Meanwhile, different sensitivity was also found to water stress created by PEG and NaCl, as demonstrated by spiny and edible amaranths [31]. In this study, *C*. *crepidioides* and *A*. *conyzoides* were more sensitive to water stress created by PEG than that by NaCl, whereas *C*. *canadensis* showed a contrasting pattern, i.e., it was more sensitive to water stress created by NaCl than that by PEG (Fig 5).

### Effects of dehydration treatment on seed viability during germination

**Fig 6:**
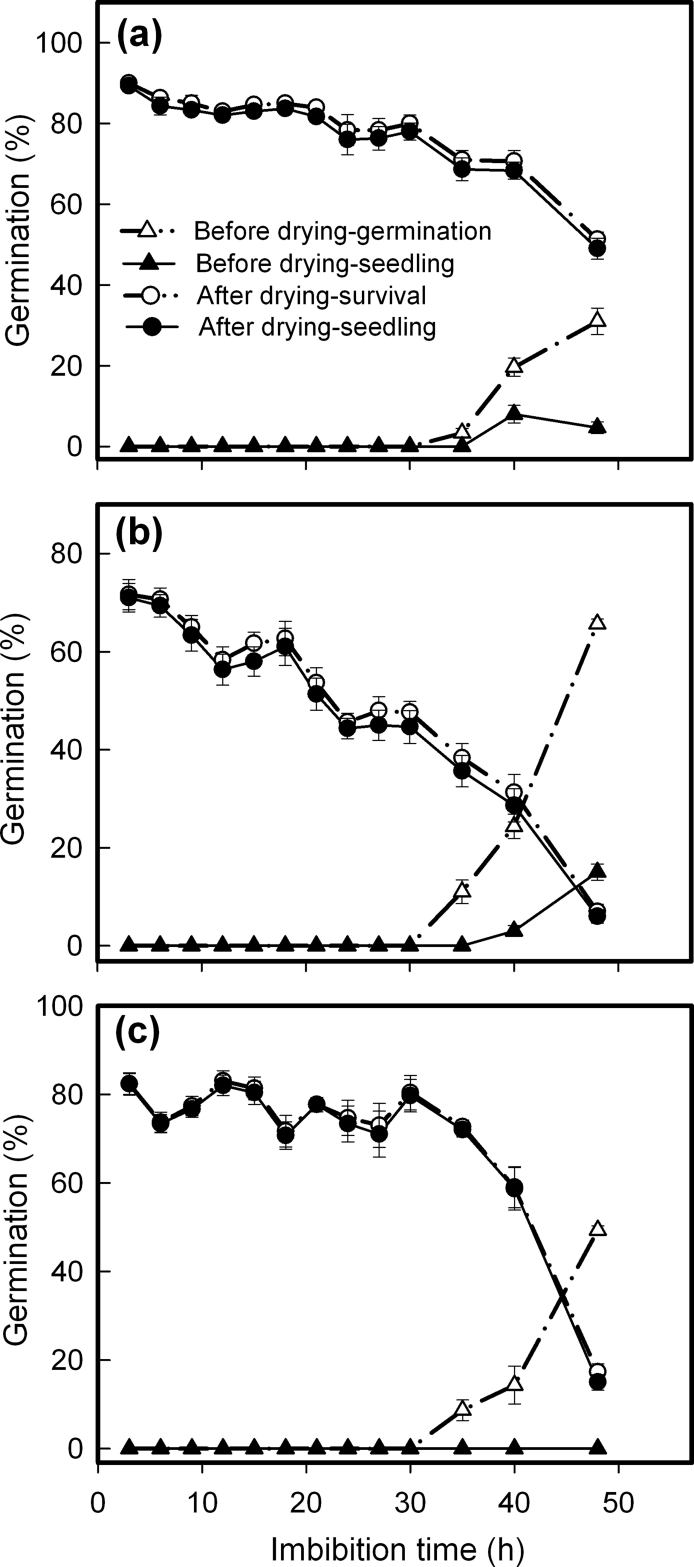
Changes in seed germination as affected by imbibition-dehydration treatment. **a.** *Crassocephalum crepidioides*; **b.** *Conyza candensis*; **c.** *Ageratum conyzoides*. After imbibition for the indicated period of time, seeds were dried for 72 h under 50% RH at 15°C, and then germinated for viability assessment. Seedling and germination or survival percentages, expressed as means±SE of six replicates of 50 seeds, were used to score germination before and after drying treatment, separately.

Imbibition duration prior to desiccation interruption is an important determinant of post-drying seed viability. Which species it was had no effect on seedling and survival percentages, but imbibition time prior to drying had a significant effect on viability (Table 1). Furthermore, *C*. *canadensis* responded to this treatment in a pattern different from the other species investigated, for its seeds lost viability linearly and quickly as imbibition duration prior to desiccation increased. For *A*. *conyzoides* seeds, imbibition for 40 h was a turning point: its seedling and survival percentage fluctuated before this point, and then decreased sharply, whereas only smaller seed viability loss was detected in *C*. *crepidioides*, and still approximately 50% seeds survived even when imbibition duration increased to 48 h. Our previous study related this trait to the seed germination rate [10]. In this study, *C*. *canadensis* seeds indeed germinated faster than *C*. *crepidioides* and *A*. *conyzoides* seeds, for example, 65% *C*. *canadensis* seeds germinated after 40 h of imbibition, whereas *C*. *crepidioides* and *A*. *conyzoides* had only 31% and 49% germination then (Fig 6).

## Discussion

The present study investigated seed germination requirements and its response to high temperature and water stress in three invasive Asteraceae weeds concurring in Xishuangbanna, SW China, and found their good adaptation to conditions in open habitats like bare grounds. These weeds all had a wide temperature range to allow seed germination, with maximum germination occurring at temperature range of 15–30°C, in accordance with findings of previous studies [3,32], only that *A*. *conyzoides* demonstrated relative preference for warmer temperatures.

These three Asteraceae species are all naturalized invasive weeds in Xishuangbanna, but widely grow in tropical and subtropical areas in the world and caused enormous economic and ecological damages. Early detection and prediction of biological invasions is regarded as the most effective measurement to reduce these damages [1]. For these reasons, scientists have been seeking for both traits of invasive plant species and that of invaded habitats since biological invasions have come into focus [33,34]. In this aspect, plant reproduction traits were found to contribute to invasiveness, including production of large number of tiny seeds [26, 31, 33], rapid germination [8,9], and germination across a wide range of environmental conditions [8,9]. However, these three Asteraceae species exhibited obvious differences in light requirement for seed germination although they all produce tiny seeds and invade open habitats. In this study *C*. *crepidioides* and *A*. *conyzoides* have photoblastic seeds, showing phytochrome-mediated germination. The ecological significance attributed to the light response in these species is that light acts as a soil depth “indicator,” allowing greater germination of surface seeds than seeds buried in soil [35]. Such examples include *Centaurea solstitialis* L.[36], *Celmisia tomentella* M. Gray & Given, and *Chrysocephalum semipapposum* (Labill.) Steetz [37], as well as *Chrysanthemum segetum* L., *Senecio jacobaea* L., and *Senecio vulgaris* L. [38]. Similar to *C*. *canadensis* reported in this study, previous studies also reported that seeds of some species can germinate highly under diverse light regimes, in both light and dark [39], such as Asteraceae *Sonchus asper* (L.) Hill [37], *Brachyscome scapigera* (Sieber ex Spreng.) DC., and *Picris angustifolia* subsp. *merxmuelleri* Lack & S. Holzapfel [38]. Bear et al. suggested that the ability to germinate in complete darkness may allow these species to establish in competitive environments by emerging through dense shrub cover [40]. We thought that whether the resultant seedling can emerge through dense shrub cover depends, but *C*. *canadensis* indeed applies a germination strategy different from *C*. *crepidioides* and *A*. *conyzoides*. Both *C*. *crepidioides* and *A*. *conyzoides* germinate only in light, so they remain quiescence in soil and wait for disturbance; while most *C*. *canadensis* seeds germinated in dark, and grow fast once disturbance occurs. It takes the advantage of going ahead over the other two species, but risk futile germination because the resultant seedling may die of starve if disturbance does not occur.

Successful biological invasions involve complex interactions between the invading species and the invaded habitats [41], for which Heger and Trepl [34] suggested a ‘key-lock models’. Located on the edge of the Asian tropics, Xishuangbanna is characterized by high temperatures, which is especially obvious on bare grounds and open habitats. Taking these into consideration, Wen et al. [26] suggested that the high temperature is a critical stress to seeds in tropical areas, where soil surface temperatures can be very high, thus high-temperature tolerance in seeds may be a requirement for them to survive in bare ground habitats. This was supported by the present study, i.e., seeds of all these three investigated species had good adaptation to high temperatures, including their tolerance to extreme high temperature of 70°C in air-dried seeds, and to continuous and daily periodical high temperature of 40°C in imbibed seeds. The same seed traits were previously reported in bamboo piper [26], Mexican sunflower [10], and spiny amaranth [31].

Furthermore, seed traits may be a vital factor to determine species distribution and preference to microhabitats. Among these three Asteraceae weeds, the distribution range of *Ageratum conyzoides* is more southern than the other two species. To support our hypothesis, *A*. *conyzoides* seeds exhibited obviously higher stress tolerance, and germinated better under higher temperature and water stress than *C*. *crepidioides* and *C*. *canadensis*, such as germination at 35°C and under daily periodical heat treatment at 40°C, and germination after continuous heat treatment at 40°C. Among them, *C*. *crepidioides* is the most sensitive to high temperature, demonstrating a rapid loss of viability after continuous heat treatment at 40°C. This is consistent with their field behavior in Xishuangbanna: *A*. *conyzoides* is the most common and troublesome among them; whereas *C*. *crepidioides* was only a moderate invader, and its invasiveness was thought to correlate with human cultivation [4]. Similarly, our previous studies compared seed high-temperature tolerance and plant invasiveness in bamboo piper [26] and Mexican sunflower [10], found that bamboo piper, whose seeds demonstrated only limited tolerance to high temperature, dominates only relatively cool and wet habitats like forest ridges and remains an moderate invader in Xishuangbanna while Mexican sunflower, whose seeds demonstrated obviously stronger tolerance to high temperature, can colonize bare ground and form monospecific stands, and demonstrates stronger invasiveness in this area [10].

We concluded that high-temperature tolerance in seeds is a prerequisite for species to colonize bare grounds and abandoned areas in tropical areas, which determines weed distribution, invasiveness and preference for microhabitats. Understanding of these interspecific differences in germination requirements and stress tolerance is useful to weed management and control. For example, from this information we could deduce that *C*. *crepidioides* more possibly invades relatively shade habitats and causes harm there whereas *A*. *conyzoides* prefers open grounds because of stronger tolerance to high temperature and water stress of its seeds, meanwhile increasing crop residue cover may help to deplete soil-stored *C*. *canadensis* seeds through promoting futile germination.
